# Supplementary material for: arfA antisense RNA regulates MscL excretory activity
Source: Life Sci Alliance. 2023 Apr 3;6(6):e202301954. doi: 10.26508/lsa.202301954 (PMC10070815; doi:10.26508/lsa.202301954)
Supplement: Supplementary file 2 [file LSA-2023-01954_TableS1.docx]

Table S1. Enrichment analysis of genomic cluster across taxa groups

| **Phyla/Class** | **Cluster** | **Cluster Count** | **Total Count** | **Percentage** |
| --- | --- | --- | --- | --- |
| *Actinobacteria* | mscl_only | 584 | 625 | 93.4 |
| *Firmicutes* | mscl_only | 470 | 608 | 77.3 |
| *Alphaproteobacteria* | mscl_only | 321 | 422 | 76.1 |
| *Bacteroidetes* | mscl_only | 262 | 286 | 91.6 |
| *Gammaproteobacteria* | no_proteins | 237 | 763 | 31.1 |
| *Betaproteobacteria* | mscl_only | 213 | 271 | 78.6 |
| *Gammaproteobacteria* | mscl_only | 173 | 763 | 22.7 |
| *Gammaproteobacteria* | distal | 137 | 763 | 18.0 |
| *Euryarchaeota* | no_proteins | 136 | 153 | 88.9 |
| *Firmicutes* | no_proteins | 135 | 608 | 22.2 |
| *Gammaproteobacteria* | proximal | 103 | 763 | 13.5 |
| *Alphaproteobacteria* | no_proteins | 100 | 422 | 23.7 |
| *Gammaproteobacteria* | arfa_only | 88 | 763 | 11.5 |
| *Epsilonproteobacteria* | no_proteins | 66 | 94 | 70.2 |
| *Tenericutes* | no_proteins | 60 | 108 | 55.6 |
| *Deltaproteobacteria* | no_proteins | 50 | 75 | 66.7 |
| *Tenericutes* | mscl_only | 48 | 108 | 44.4 |
| *Crenarchaeota* | no_proteins | 48 | 48 | 100.0 |
| *Actinobacteria* | no_proteins | 41 | 625 | 6.6 |
| *Spirochaetes* | no_proteins | 41 | 48 | 85.4 |

Note: Subset of taxonomic groups shown, only those containing >1% of total number of genomes present. See Data S1 for genomic and taxonomic data.
