# Supplementary material for: arfA antisense RNA regulates MscL excretory activity
Source: Life Sci Alliance. 2023 Apr 3;6(6):e202301954. doi: 10.26508/lsa.202301954 (PMC10070815; doi:10.26508/lsa.202301954)
Supplement: Supplementary file 3 [file LSA-2023-01954_TableS2.docx]

**Table S2 Syntenic analysis of *arfA* proximal and overlapping genes**

| **NCBI protein annotation** | **Total count** | **Average ArfA Bit score ± SD** | **Genera** | **ArfA-only count** | **Distal count** |
| --- | --- | --- | --- | --- | --- |
| iron-sulfur cluster assembly protein IscA | 10 | 75.7 ± 2.7 | *Neisseria* | 4 | 6 |
| threonine/serine exporter family protein | 9 | 84.1± 2.5 | *Vibrio* | 9 | 0 |
| 30S ribosomal protein S12 methylthiotransferase RimO | 9 | 83.0 ± 4.2 | *Shewanella* | 9 | 0 |
| hypothetical protein | 6 | 70.8 ± 22.5 | *multiple* | 3 | 3 |
| Trk system potassium transporter TrkA | 4 | 89.5 ± 2.2 | *Xenorhabdus* | 4 | 0 |
| dethiobiotin synthase | 4 | 77.8 ± 3.8 | *Mannheimia* | 2 | 2 |
| cytochrome b | 3 | 73.3 ± 0.3 | *Pseudoalteromonas* | 3 | 0 |
| radical SAM family heme chaperone HemW | 3 | 83.2 ± 4.1 | *Aeromonas* | 0 | 3 |
| diguanylate cyclase | 2 | 84.9 ± 2.1 | *Shewanella,*  *Pseudoalteromonas* | 2 | 0 |
| pyrimidine 5'-nucleotidase | 2 | 82.5 ± 0 | *Agarivorans* | 2 | 0 |
| DUF3465 domain-containing protein | 2 | 82.1 ± 1.6 | *Shewanella* | 2 | 0 |
| helix-turn-helix domain-containing protein | 2 | 88.7 ± 6.7 | *Leminorella, Pragia* | 0 | 2 |
| oxygen-dependent coproporphyrinogen oxidase | 1 | 73 | *Colwellia* | 1 | 0 |
| FNR family transcription factor | 1 | 82.2 | *Frederiksenia* | 0 | 1 |
| amino acid ABC transporter substrate-binding protein | 1 | 85.8 | *Shewanella* | 1 | 0 |
| YqaE/Pmp3 family membrane protein | 1 | 78.7 | *Dongshaea* | 1 | 0 |
| response regulator transcription factor | 1 | 53 | *Halomonas* | 0 | 1 |
| PAS domain-containing methyl-accepting chemotaxis protein | 1 | 83.8 | *Vibrio* | 1 | 0 |
| diacylglycerol kinase | 1 | 80.8 | *Vibrio* | 1 | 0 |
| recombinase RecA | 1 | 78.9 | *Paralysiella* | 0 | 1 |
| sulfate transporter CysZ | 1 | 87.6 | *Haemophilus* | 0 | 1 |
| NCS2 family permease | 1 | 35.1 | *Cardiobacterium* | 0 | 1 |
| methyltransferase domain-containing protein | 1 | 78.2 | *Pasteurella* | 0 | 1 |
| methylenetetrahydrofolate reductase | 1 | 75.1 | *Bibersteinia* | 0 | 1 |
| N-acetyltransferase | 1 | 85 | *Plesiomonas* | 0 | 1 |
| IS982 family transposase | 1 | 88.7 | *Xenorhabdus* | 1 | 0 |
| LacI family DNA-binding transcriptional regulator | 1 | 73 | *Pseudoalteromonas* | 1 | 0 |

Genes located within 110 nucleotides from *arfA* were included. See Data S1 for genomic and taxonomic data.
