# Supplementary material for: arfA antisense RNA regulates MscL excretory activity
Source: Life Sci Alliance. 2023 Apr 3;6(6):e202301954. doi: 10.26508/lsa.202301954 (PMC10070815; doi:10.26508/lsa.202301954)
Supplement: Supplementary file 4 [file LSA-2023-01954_TableS3.docx]

## Table S3. E. coli strains and plasmids used in this study.

| **Strain or Plasmid** | **Description** | **Reference or source** |
| --- | --- | --- |
| **Strain** |  |  |
| BL21 (DE3)   - Wild-type - Wild-type *mscLHis* - Δ*arfA* *mscLHis* | - Replacement of wild-type *mscL* with *mscLHis* gene tagged with hexahistidine at C-terminus. Containing kanamycin resistance cassette - A knockout mutant of *arfA*. Deletion of transcription starting site (TSS) and first 43 amino acids of ArfA (1-43aa). Replacement of wild-type *mscL* with *mscLHis* gene tagged with hexahistidine at C-terminus. Containing kanamycin resistance cassette | - Novagen - This study - This study |
| K-12 MG1655   - Wild-type - Δ*arfA* - Δ*mscL* | - A knockout mutant of *arfA*. Deletion of transcription starting site (TSS) and first 43 amino acids ArfA (1-43aa). Containing kanamycin resistance cassette. - A knockout mutant of *mscL*. Deletion of the promoter, 5’UTR and CDS of *mscL*. Containing kanamycin resistance cassette. | - Lab stock - This study - This study |
| K-12 BW25113   - Δ*rpoS* - Δ*smpB* | - A knockout mutant of *rpoS*. Containing kanamycin resistance cassette - A knockout mutant of *smpB*. Containing kanamycin resistance cassette | - Keio collection (1) |
| MC1061   - ∆*rnc* | - A non-functional mutant of RNAseIII (∆*rnc-*38)   Containing kanamycin resistance cassette | - (2) |
| **Plasmid** |  |  |
| p131B   - p131B_P*_mscL_*-sfGFP - p131B_P*_arfA_*-sfGFP | - p131B plasmid cloned with *mscL* promoter fused with *sfGFP* reporter (P*_mscL_*-sfGFP) as a construct to study promoter activity. Ampicillin or carbenicillin resistance - p131B plasmid cloned with *arfA* promoter fused with *sfGFP* reporter (P*_arfA_*-sfGFP) as a construct to study promoter activity. Ampicillin or carbenicillin resistance | - Lab stock - This study - This study |
| pET44   - pET44-sfGFP | - pET44 (expression plasmid) cloned with *sfGFP* for recombinant protein overexpression (IPTG inducible) and monitoring recombinant protein excretion during osmotic and translational stress conditions. Ampicillin or carbenicillin resistance | - Novagen - This study |
| pSIM18 | - Expressing λ-red recombinase for homologous recombination of target DNA into *E. coli* genome. Used to integrate (recombination) Kan resistance cassette for knockout mutation of *arfA* (Δ*arfA*) and *mscL* (Δ*mscL*) in K-12 MG1655, and to integrate *mscLHis*-Kan resistance cassette for *mscL* replacement with *mscLHis* (wild-type) and along with knockout mutation of arfA (Δ*arfA*) in BL21 (DE3). Hygromycin resistance | - A gift from SynBioChem, University of Manchester (3) |
| pRL128 | - Used to generate Kan-resistance cassette for chromosome engineering (knockout and gene replacement) | - Lab stock |
| pCA24N   - p*FL* - p*Δ(154-216nt)* - p*IL* - p*A18T* - p*sRNA* | - Expression plasmid. T5-based promoter for recombinant gene expression (IPTG inducible). Gentamycin resistance. Used to generate rescue strain (plasmid expression) for gene expression study - CDS of *arfA* gene cloned in pCA24N - CDS of *arfA* gene missing fragment from nucleotide 154 o 216 cloned in pCA24N - CDS of *arfA* gene containing inverted repeats at nucleotide position   147 (G into A), 156 (G into A), 159 (C into A), 162 (T into C) and  171 (A into G) cloned in pCA24N   - CDS of *arfA* gene containing the substitution of the amino acid Alanine (AGC) in position 18 with a Threonine (ACC) cloned in pCA24N - Fragment from nucleotide 160 to 203 from *arfA* CDS gene cloned in pCA24N | - Lab stock - This study - This study - This study - This study - This study |

**References**

1. Baba, T., Ara, T., Hasegawa, M., Takai, Y., Okumura, Y., Baba, M., Datsenko, K.A., Tomita, M., Wanner, B.L. and Mori, H. (2006) Construction of Escherichia coli K-12 in-frame, single-gene knockout mutants: the Keio collection. *Mol Syst Biol*, **2**, 2006 0008.

2. Babitzke, P., Granger, L., Olszewski, J. and Kushner, S.R. (1993) Analysis of mRNA decay and rRNA processing in Escherichia coli multiple mutants carrying a deletion in RNase III. *J Bacteriol*, **175**, 229-239.

3. Chan, W., Costantino, N., Li, R., Lee, S.C., Su, Q., Melvin, D., Court, D.L. and Liu, P. (2007) A recombineering based approach for high-throughput conditional knockout targeting vector construction. *Nucleic Acids Res*, **35**, e64.
