# Supplementary material for: arfA antisense RNA regulates MscL excretory activity
Source: Life Sci Alliance. 2023 Apr 3;6(6):e202301954. doi: 10.26508/lsa.202301954 (PMC10070815; doi:10.26508/lsa.202301954)
Supplement: Supplementary file 5 [file LSA-2023-01954_TableS4.docx]

## Table S4. Primers used for cloning and generation of targeted gene deletion strains (source IDT)

| **Primer set** | **Sequence** | **Use** |
| --- | --- | --- |
| arfA_Δ*(154-216nt)* F  + | TAACTATGCGGCCGCTAAGGG | Used to generate p*Δ(154-216nt)* by PCR mutagenesis of p*FL* |
| arfA_Δ (154-216nt) R  H1_Δ(P-44) *mscL*  +  H2_*mscL*  H2_Δ (TSS)*arfA*  +  H1_*arfA*  H1_Δ(P-44) *mscL* V2  H1_*smpB*  +  H2_*smpB* | P-CCAGTTCCCCCGATTGCC  CTAATGACGCCTTATTATTTCCCTTTGATTATCAAGGATTAATTAAATTC*GAGGTGTAGGCTGGAGCTGC*  ACCACTGGTCTTCTGCTTTCAGGCGCTTGTTAAGAGCGGTTATTCTGCT*CTTGGGATCCGTCGACCTGCA*  CTTGAACAAGGGGCGAGTGGCGTTAAGAGTGGTTGTTGATTTTTTGCACT*GAGGTGTAGGCTGGAGCTGC*  ACCAGTGGTAAAAAAGTGATTTACTTTCTTGCCACTGGCCTCCCAGTTC*C TTGGGATCCGTCGACCTGCA*  CTAATGACGCCTTATTATTTCCCTTTGATTATCAAGGATTAATTAAATTC*TTGGGATCCGTCGACCTGCA*  GATATGGGGTGTTTTCGATTTCAGATTACCGATGATTCACGACGCTTATG*TTGGGATCCGTCGACCTGCA*  AGGAACTGGTCAATAATTGGAGTGCAGGTTTAACGGTGGGCGTTTTTCAT*GAGGTGTAGGCTGGAGCTGC* | Used to amplify the FRT-flanked kanamycin selection cassettes by PCR to generate Δ*mscL* strains  Used to amplify the FRT-flanked kanamycin selection cassettes by PCR to generate Δ*arfA* strains  Used to amplify, with H2_Δ (TSS)*arfA*, FRT-flanked kanamycin selection cassettes by PCR to generate Δ*mscL*Δ*arfA* strains  Used to amplify FRT-flanked kanamycin selection cassettes by PCR to generate Δ*smpB* strain |
