# Supplementary material for: arfA antisense RNA regulates MscL excretory activity
Source: Life Sci Alliance. 2023 Apr 3;6(6):e202301954. doi: 10.26508/lsa.202301954 (PMC10070815; doi:10.26508/lsa.202301954)
Supplement: Supplementary file 6 [file LSA-2023-01954_TableS5.docx]

## Table S5. Primers used for qRT-PCR (source IDT)

| **Primer name** | **Sequence** | **Use amplicon size (nt)** | **%E (F+R)** | **E (F+R)** | **Slope** |
| --- | --- | --- | --- | --- | --- |
| *msc*L F | TGCCTCCTCTGGGCTTATTA | *mscL* transcript quantification 96 | 110 | 2.111 | -3.08 |
| *msc*L R | GCATCACAACAGCAGGGATA | Locus_Tag b3291 |  |  |  |
| *arf*A F | TCAGCATACTAAAGGGCAGATAAA | *arfA* transcript quantification 76 | 98 | 1.939 | -3.38 |
| *arf*A R | CTACGCGCTGTCGGAATAA | Locus_Tag b4550 |  |  |  |
| *rec*A F | TACCGGTTCGCTTTCACTG | *recA* transcripts quantification 76 | 99 | 2.038 | -3.23 |
| *rec*A R | TCCGTAGATTTCGACGATACG | Locus_Tag b2699 |  |  |  |
| *cys*G F | CACTGCGTACCCCAGGAA | *cysG* transcripts quantification (4) | 99 | 2.011 | -3.29 |
| *cys*G R | TTTGCCTTTTTGCGCTTC | Locus_Tag b3368 |  |  |  |
| *M*1 F | GAAGCTGACCAGACAGTCGC | *M1* transcripts quantification (5) |  |  |  |
| *M*2 R | AGGTGAAACTGACCGATAAG | Locus_Tag b3123 |  |  |  |

**References**

4. Zhou, K., Zhou, L., Lim, Q., Zou, R., Stephanopoulos, G. and Too, H.P. (2011) Novel reference genes for quantifying transcriptional responses of Escherichia coli to protein overexpression by quantitative PCR. *BMC Mol Biol*, **12**, 18.

5. Boram Lim, K.L. (2015) Stability of the Osmoregulated Promoter-Derived proP mRNA Is Posttranscriptionally Regulated by RNase III in Escherichia coli. *Journal of Bacteriology*, **197**, 1297-1305.
